# Omega-shaped epiglottis: a challenge

**DOI:** 10.1016/j.bjane.2021.02.038

**Published:** 2021-03-21

**Authors:** Joana Veiga, Cristina Gomes

**Affiliations:** Hospital de Braga E.P.E, Braga, Portugal

A heavy smoker 49-year-old man, American Society of Anestesiologists (ASA) physical status III, with positive history of gradually worsening dyspnea was proposed for Suspension Microlaryngoscopy surgery. Airway evaluation showed a grade-III Mallampati score and no apparent or palpable cervical mass. After preoxygenation and induction, orotracheal intubation was performed with C-MAC D blade Videolaryngoscope®, and founded an omega-shaped epiglottis (OSE) (Fig. 1), with vocal cords visualized only after lifting the epiglottis with the tip of the curved blade (Fig. 2). Intubation was accomplished using a 4.0- mm cuffed microlaryngeal orotracheal tube, anesthesia was maintained with sevoflurane and controlled ventilation. Anesthesia emergency was uneventful.

**Figure 1:**
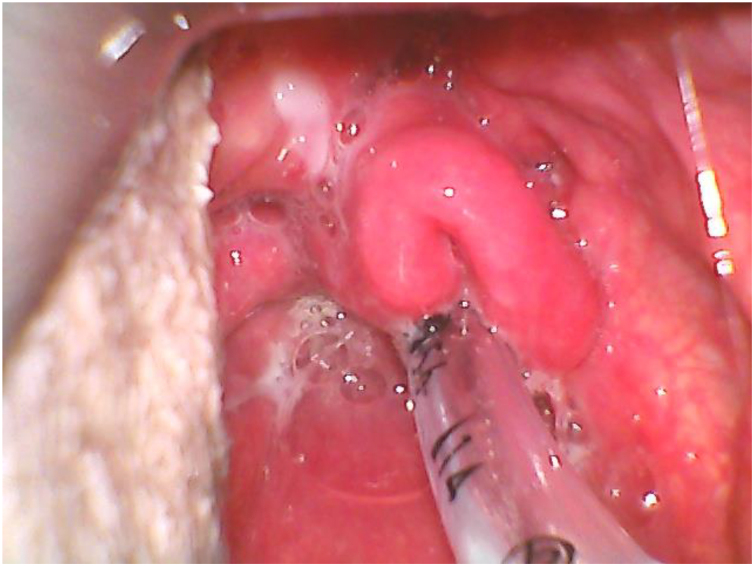
Omega-shaped epiglottis visualized with C-MAC D Blade videolaryngoscope.

**Figure 2:**
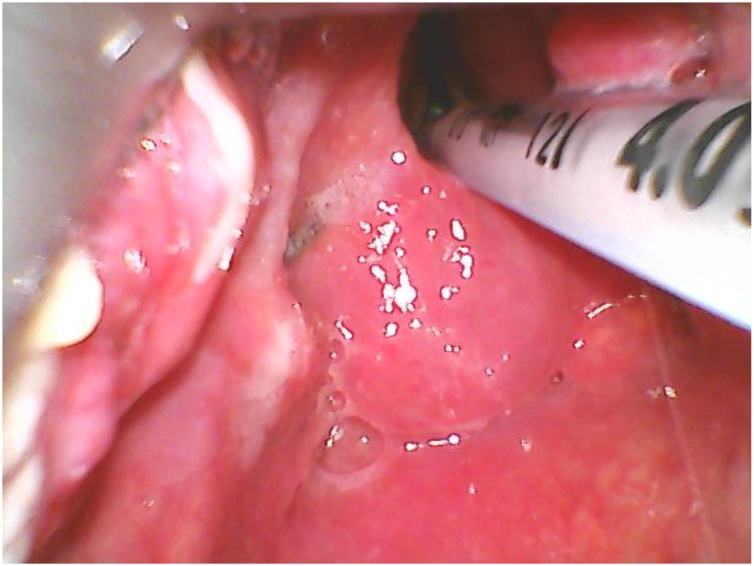
Exposition of larynx and vocal cords after lifting epiglottis.

OSE is a variant configuration of epiglottis in which the lateral folds are curled inwards.^1^ Although not necessarily pathological, it may be associated with laryngomalacia and supraglottitis.^2^ From the anesthetic point of view, potential problems of OSE include variable airway obstruction and compromise. Anatomical changes of the epiglottis should serve as a warning for difficult airway, namely with facemask ventilation and orotracheal intubation/extubation.^3^ Preoperative anesthetic evaluation should include investigation symptoms suggestive of intermittent airway obstruction and image evaluation (MRI or CT-scan of the head and neck) (Fig. 3).

**Figure 3:**
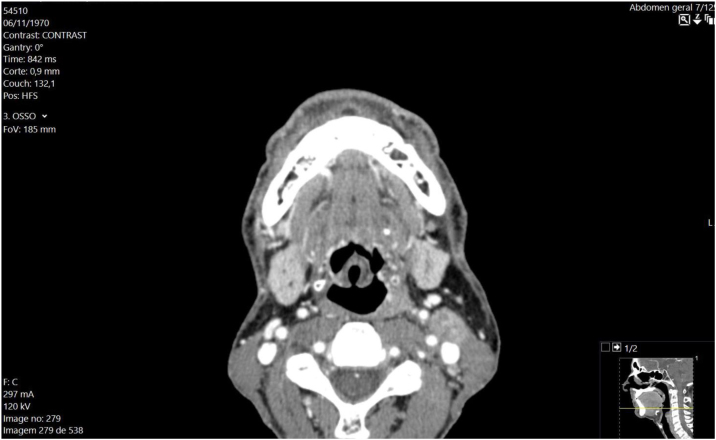
CT-Scan of head and neck showing omega epiglottis.

## References

[1] Ayari S., Aubertin G., Girschig H. (2012). Pathophysiology and diagnostic approach to laryngomalacia in infants. Eur Ann Otorhinolaryngol Head Neck Dis..

[2] Kovacs A., Haran S., Paddle P. (2019). Chronic non-granulomatous supraglottitis of a male adolescent and its successful management with azathioprine. BMJ Case Reports CP..

[3] Laschat M., Kaufmann J., Wappler F. (2016). Laryngomalacia with Epiglottic Prolapse Obscuring the Laryngeal Inlet. Anesthesiology..

